# [^89^Zr]-CD8 ImmunoPET imaging of glioblastoma multiforme response to combination oncolytic viral and checkpoint inhibitor immunotherapy reveals CD8 infiltration differential changes in preclinical models

**DOI:** 10.7150/thno.89206

**Published:** 2024-01-01

**Authors:** Carlos A. Gallegos, Yun Lu, Jennifer C. Clements, Patrick N. Song, Shannon E. Lynch, Alessandro Mascioni, Fang Jia, Yolanda E. Hartman, Adriana V. F. Massicano, Hailey A. Houson, Suzanne E. Lapi, Jason M. Warram, James M. Markert, Anna G. Sorace

**Affiliations:** 1 Department of Biomedical Engineering, University of Alabama at Birmingham, Birmingham, AL, USA.; 2 Department of Radiology, University of Alabama at Birmingham, Birmingham, AL, USA.; 3 Graduate Biomedical Sciences, University of Alabama at Birmingham, Birmingham, AL, USA.; 4 Department of Neurosurgery, University of Alabama at Birmingham, Birmingham, AL, USA.; 5 Imaginab, Inc, Inglewood, CA, USA.; 6 Department of Chemistry, University of Alabama at Birmingham, Birmingham, AL, USA.; 7 O'Neal Comprehensive Cancer Center, University of Alabama at Birmingham, Birmingham, AL, USA.; 8 Department of Otolaryngology, University of Alabama at Birmingham, Birmingham, AL, USA.

**Keywords:** glioblastoma stem cell, cytotoxic T cell, ImmunoPET, molecular imaging, oncolytic herpes simplex virus

## Abstract

**Rationale:** Novel immune-activating therapeutics for the treatment of glioblastoma multiforme (GBM) have shown potential for tumor regression and increased survival over standard therapies. However, immunotherapy efficacy remains inconsistent with response assessment being complicated by early treatment-induced apparent radiological tumor progression and slow downstream effects. This inability to determine early immunotherapeutic benefit results in a drastically decreased window for alternative, and potentially more effective, treatment options. The objective of this study is to evaluate the effects of combination immunotherapy on early CD8^+^ cell infiltration and its association with long term response in orthotopic syngeneic glioblastoma models.

**Methods:** Luciferase positive GBM orthotopic mouse models (GSC005-luc) were imaged via [^89^Zr]-CD8 positron emission tomography (PET) one week following treatment with saline, anti-PD1, M002 oncolytic herpes simplex virus (oHSV) or combination immunotherapy. Subsequently, brains were excised, imaged via [^89^Zr]-CD8 ImmunoPET and evaluated though autoradiography and histology for H&E and CD8 immunohistochemistry. Longitudinal immunotherapeutic effects were evaluated through [^89^Zr]-CD8 PET imaging one- and three-weeks following treatment, with changes in tumor volume monitored on a three-day basis via bioluminescence imaging (BLI). Response classification was then performed based on long-term BLI signal changes. Statistical analysis was performed between groups using one-way ANOVA and two-sided unpaired T-test, with p < 0.05 considered significant. Correlations between imaging and biological validation were assessed via Pearson's correlation test.

**Results:** [^89^Zr]-CD8 PET standardized uptake value (SUV) quantification was correlated with *ex vivo* SUV quantification (r = 0.61, p < 0.01), autoradiography (r = 0.46, p < 0.01), and IHC tumor CD8^+^ cell density (r = 0.55, p < 0.01). Classification of therapeutic responders, via bioluminescence signal, revealed a more homogeneous CD8^+^ immune cell distribution in responders (p < 0.05) one-week following immunotherapy.

**Conclusions:** Assessment of early CD8^+^ cell infiltration and distribution in the tumor microenvironment provides potential imaging metrics for the characterization of oHSV and checkpoint blockade immunotherapy response in GBM. The combination therapies showed enhanced efficacy compared to single agent immunotherapies. Further development of immune-focused imaging methods can provide clinically relevant metrics associated with immune cell localization that can inform immunotherapeutic efficacy and subsequent treatment response in GBM patients.

## Introduction

Glioblastoma multiforme (GBM) is the most common central nervous system (CNS) malignancy, and its diagnosis is associated with poor prognosis and high recurrence despite standard clinical interventions 1-4. There has been a growing interest in the use of immunotherapies, including virotherapy and immune checkpoint inhibitors (ICI), for the treatment of GBM due to their potential for improved survival and long-term cancer remission through immune surveillance 3, 5-7. In particular, oncolytic virotherapy has been associated with selective targeting of cancer cells and promotion of antitumoral immunological responses given its inherent viral immunogenicity along with the release of tumor antigens following cancer cell lysis 3, 8. Treatment with M002, a genetically engineered oncolytic herpes simplex virus (oHSV) expressing murine interleukin-12 (IL-12), has been shown to prolong survival in preclinical GBM and other cancer models 4, 9-12. Additionally, combining oHSV with ICI, such as those targeting the Program Cell Death Protein-1 (anti-PD1), has shown greater antitumor activity and potential improvement of immunological memory in murine preclinical models 13. Nevertheless, similar to other immunotherapies, responses in oHSV and combination therapies in GBM are variable, with a subset of the treated population not exhibiting long-term remission 13, 14.

Immunotherapy-induced effects, which primarily consist of intratumoral immune cell infiltration and localized inflammation, have been associated with early progressive radiographic findings in standard GBM assessment through magnetic resonance imaging (MRI) 15-17. Given these considerations, current guidelines, under the immunotherapy Response Assessment for Neuro-Oncology (iRANO), recommend performing follow-up imaging as far as six months from initial treatment to fully differentiate true tumor progression and determine clinical benefit 7, 18. In addition, prognostic application of standard molecular imaging through 2-deoxy-2[^18^F]-fluoro-D-glucose (FDG) positron emission tomography (PET) has been limited given the natural high [^18^F]-FDG uptake in normal brain and activated inflammatory cells, decreasing specificity for the target lesions 19-21. This has led to a need to better characterize the biological interactions and identify predictive metrics associated with effective response during single agent and combination immunotherapies in brain tumors.

Immune cell populations associated with cytotoxic activity on cancer cells have become an appealing target for the monitoring of immunotherapy responses. Cytotoxic CD8^+^ T cells, through the release of effector molecules (i.e. cytokines, perforin and granzyme B), play an essential role on anticancer immune response 22. Further, increased CD8^+^ T cell infiltration in the tumor microenvironment has been associated with positive treatment response and improved prognosis in multiple preclinical cancer models 23, 24 and clinical trials 25-27. As a biomarker, non-invasive ImmunoPET imaging agents targeting CD8^+^ cell populations have been explored for the evaluation of long-term response to immunotherapy, highlighting their ability to distinguish treatment responsive tumors in preclinical studies 24, 28-30. In addition, evaluations using a CD8-targeting ImmunoPET minibody have shown its capability to monitor intratumoral CD8^+^ cells and potential to predict early response in preclinical studies 30. Furthermore, early ImmunoPET evaluation of CD8^+^ cell populations in GBM have demonstrated the ability of this approach to monitor CD8^+^ cell infiltration following immunotherapy in preclinical immunocompetent 31 and humanized models 32. Clinical translation of CD8-targeted ImmunoPET imaging using a minibody targeting agent, [^89^Zr]-Df-IAB22M2C, has been further shown to be safe and have favorable kinetics for early monitoring of CD8-rich tissues in cancer patients 33, 34. Nevertheless, there are limited studies focusing on the longitudinal downstream effects of CD8^+^ changes in immune response and infiltration following oHSV immunotherapy in preclinical glioma.

In these studies, we explored *in vivo* and *ex vivo* CD8 ImmunoPET imaging for the noninvasive monitoring of CD8^+^ cells under single agent and combination M002 oHSV and anti-PD1 immunotherapy in preclinical GBM models. In addition, we evaluated long-term immunotherapy response and its association with CD8 ImmunoPET imaging findings. To our knowledge, CD8 ImmunoPET has not been evaluated under combination oHSV and anti-PD1 immunotherapies, as well as a long-term metric for immunotherapy response, in GBM models. Mice with orthotopic GBM tumors (GSC005-luc) were imaged with [^89^Zr]-CD8 minibody ImmunoPET *in vivo* and *ex vivo*, for early immune cell monitoring, with further longitudinal response assessment being performed. Biological validation, through autoradiography and immunohistochemistry (IHC), was performed to corroborate imaging findings. Non-invasive monitoring of cytotoxic immune cells provides clinically relevant information on early novel combinational immunotherapy effects and has the potential to serve as a diagnostic approach for response in GBM patients.

## Results

### [^89^Zr]-CD8 ImmunoPET revealed differences in brain CD8^+^ cell infiltration across immunotherapy groups

**Figure 1:**
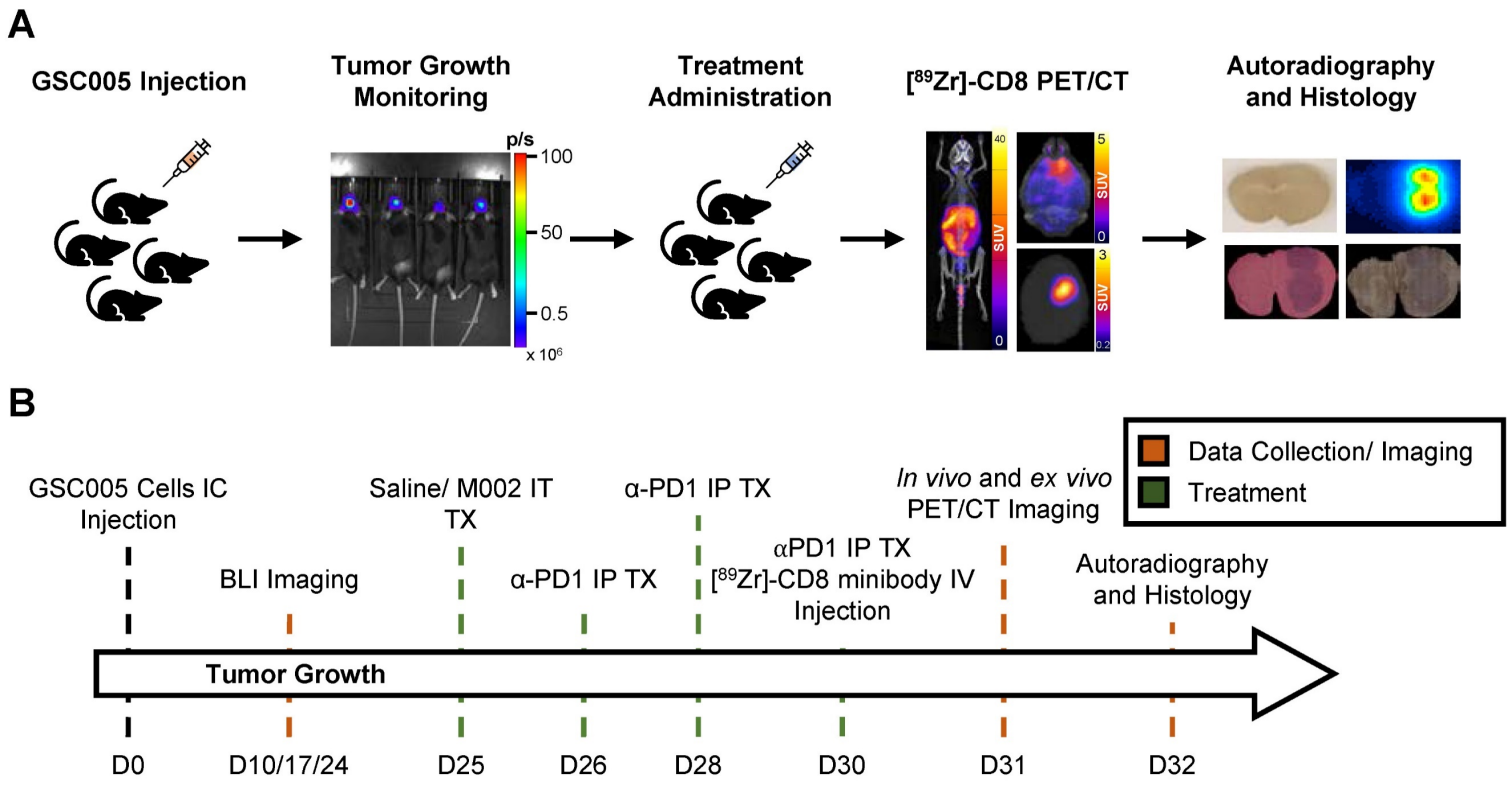
** Representative schematic for CD8^+^ cell infiltration assessment experiment. (A)** Representative images of key steps for the experimental design evaluating GSC005-luc GBM tumor model under single agent and combination anti-PD1 and M002 oHSV immunotherapy with [^89^Zr]-CD8 ImmunoPET imaging, autoradiography, and histological analysis. (B) Experimental timeline. GSC: Glioblastoma Stem Cells, IC: intracranial, BLI: bioluminescence, IT: intratumoral, TX: treatment, IP: intraperitoneal, Zr: Zirconium, IV: Intravenous.

**Figure 2:**
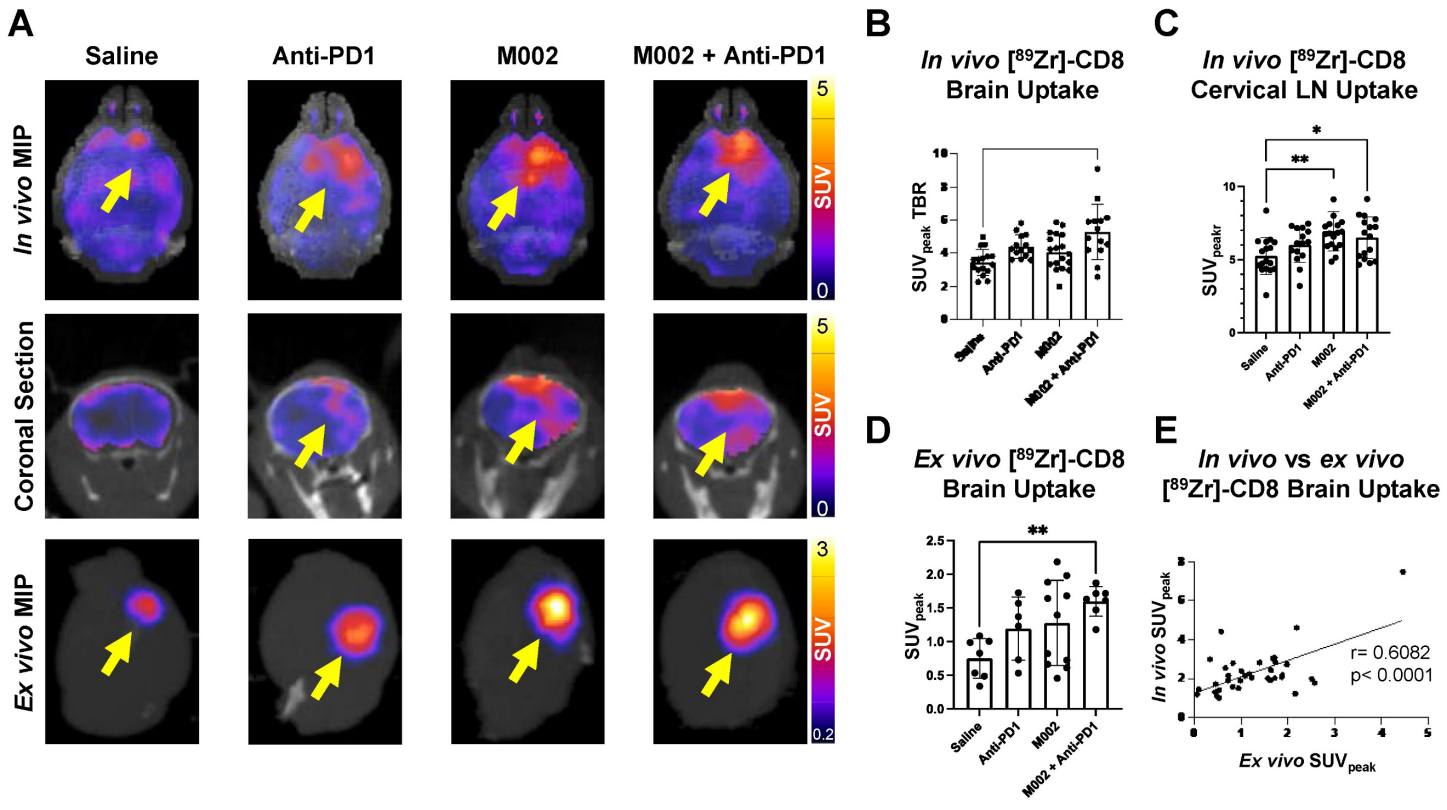
** [^89^Zr]-CD8 ImmunoPET imaging revealed increased CD8^+^ T cell infiltration in combination M002 and anti-PD1 immunotherapy. (A)** Representative *in vivo* [^89^Zr]-CD8 minibody ImmunoPET brain MIP (top row), coronal head cross section (middle row) and *ex vivo* brain MIP (bottom row). Signal uptake can be seen on regions of tumor implantation as pointed by the yellow arrows on each representative image. **(B)** Quantification of SUV_peak_ of the right side of the brain normalized to the contralateral brain SUV_mean_ (SUV_peak_ TBR), from short-term and longitudinal cohorts, showed a significant increase in CD8^+^ infiltration in the combination oHSV and anti-PD1 group (p < 0.0001). **(C)** SUV_peak_ of cervical lymph nodes normalized to blood revealed increased CD8^+^ cell presence in single agent oHSV (p < 0.01) and combination immunotherapy (p < 0.05) groups relative to controls. **(D)** SUV_peak_ of the *ex vivo* whole brain region demonstrated a significant difference between combination the immunotherapy and controls (p < 0.01). **(E)** *In vivo* SUV_peak_ was correlated with *ex vivo* SUV_peak_ (r = 0.61, p < 0.0001). MIP: Maximum Intensity Projection, LN: Lymph Nodes.

Intratumoral CD8^+^ T cell infiltration was explored one week following immunotherapy administration through *in vivo* and *ex vivo* [^89^Zr]-CD8 minibody ImmunoPET imaging, see Figure 1A and 1B for graphical overview and experimental timeline. As shown on Figure 2A, CD8^+^ cell infiltration was primarily localized to the tumor region and was visualized on both *in vivo* and *ex vivo* ImmunoPET. Average *in vivo* peak standardized uptake value (SUV_peak_) tumor to background ratios (TBR) within groups showed increased CD8 infiltration in the tumor in combination M002 oHSV and anti-PD1 (5.29 ± 1.66, p < 0.0001) when compared to saline controls (3.46 ± 0.77), see Figure 2B. In addition, CD8^+^ cell presence on the cervical lymph nodes, as measured by SUV_peak_ normalized to blood was increased in single agent M002 oHSV (6.93 ± 1.34, p = 0.001) and combination immunotherapy (6.51 ± 1.42, p = 0.02) groups when compared to controls (5.26 ± 1.27), see Figure 2C. *Ex vivo* SUV_peak_ values were found to be consistent with *in vivo* analysis, with increased [^89^Zr]-CD8 minibody tumor uptake in the combination treated group (1.60 ± 0.22, p < 0.01) and trending towards increases in single agent M002 (1.19 ± 0.47, p = 0.06) and anti-PD1 (1.28 ± 0.63, p = 0.06) when compared to controls (0.75 ± 0.30), see Figure 2D. Finally, as shown in Figure 2E, SUV_peak_ across *in vivo* and *ex vivo* scans were significantly correlated (r = 0.61, p < 0.0001), providing support for the proper quantification of the immuno-targeted tracer uptake on the brain *in vivo*. See Table S1 for numerical data associated with these results.

### [^89^Zr]-CD8 ImmunoPET revealed longitudinal changes in intracranial CD8^+^ T cell localization over time

**Figure 3:**
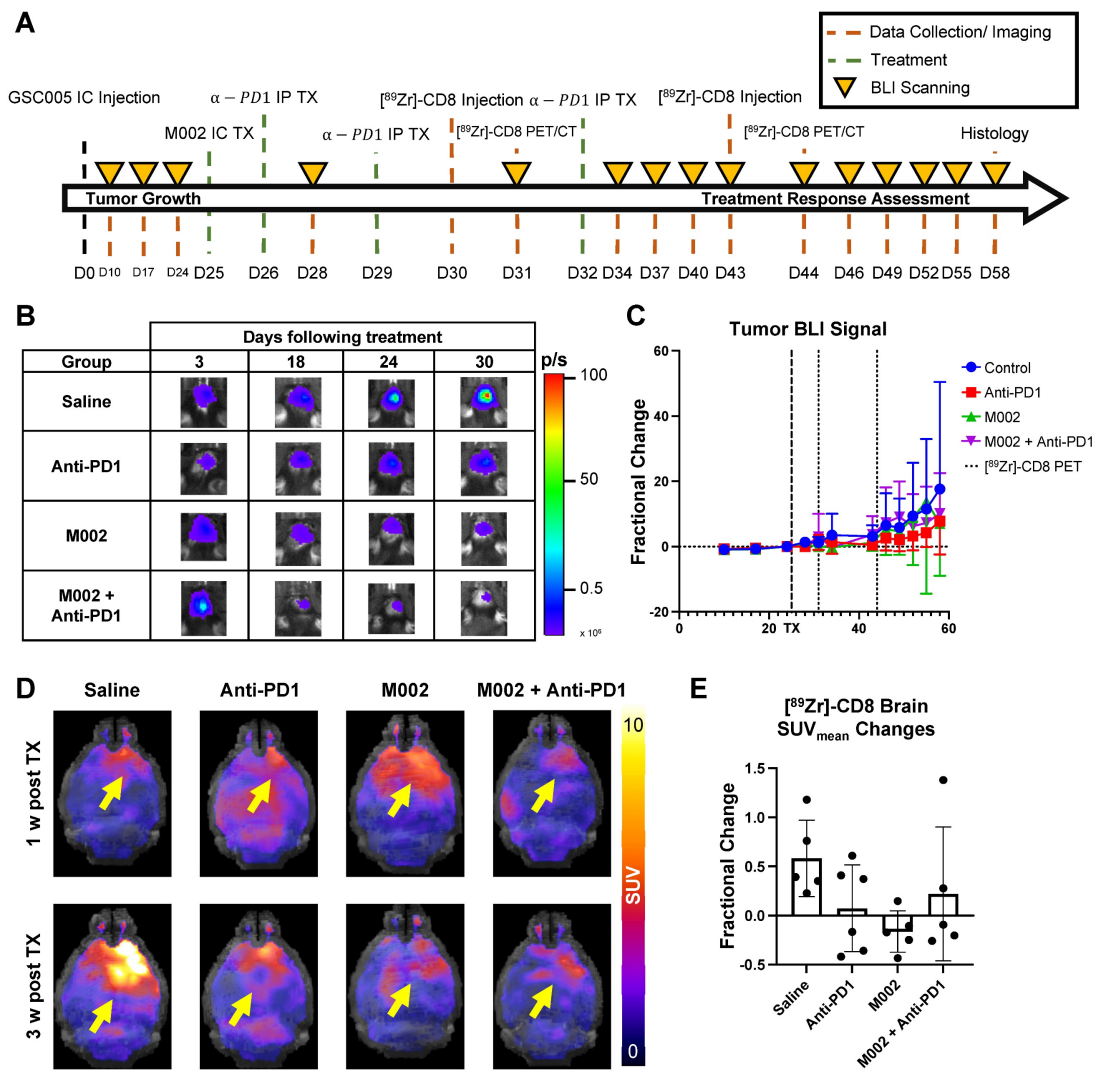
** Longitudinal immunotherapy response assessment through bioluminescence and [^89^Zr]-CD8 ImmunoPET imaging revealed changes in tumor volume and immune cell infiltration in response to therapy. (A)** Representative schematic for longitudinal immunotherapy response assessment experimental timeline. This experiment followed GSC005-luc tumors longitudinally under immunotherapy treatment through BLI and [^89^Zr]-CD8 minibody ImmunoPET/CT imaging. **(B)** Representative bioluminescence scans, showing luciferase signal emitted from the tumor region in each therapy group through the course of the experiment, demonstrated an increase in tumor burden in the control group and variable response in the treatment groups. **(C)** Fractional changes (mean +/- stdev) from baseline of mean bioluminescence signal in are shown for each group. **(D)** Representative *in vivo* [^89^Zr]-CD8 minibody ImmunoPET/CT brain MIP images one week (top row) and three weeks (bottom row) following treatment. Signal accumulation on the tumor region pointed by the yellow arrow. **(E)** Brain SUV_mean_ fractional change across one- and three-week imaging showed trending significant decreases in CD8^+^ cell accumulation in the brain on single agent M002 group (p = 0.052) relative to control. Quantification showed variability in changes in CD8^+^ cell presence on immunotherapy groups. BLI: Bioluminescence Imaging, TX: Treatment.

Intratumoral CD8^+^ cell concentration was evaluated one- and three-weeks following combination and single agent immunotherapies through [^89^Zr]-CD8 minibody ImmunoPET imaging, see Figure 3A and 3D for longitudinal experimental timeline and representative scans. Evaluation of brain CD8^+^ cell presence, as quantified by SUV_mean_, between the one- and three-week imaging timepoints showed an increase of 58.2 ± 38.9% in controls, 7.4 ± 44.1% in anti-PD1 (p = 0.20), and 22.1 ± 68.0% (p = 0.48) in combination group, with a decrease of 16.2 ± 21.2% (p = 0.05) in single agent M002. Of interest, all control mice showed an overall increase in whole brain CD8 presence on the third week imaging when compared to the first, while immunotherapy treated groups showed variability in localization, with the majority exhibiting a decrease in CD8^+^ cell presence (N = 3/6 anti-PD1, 4/5 M002, 3/5 combination), see Figure 3E.

### Bioluminescence imaging permitted classification of immunotherapy responders

**Figure 4:**
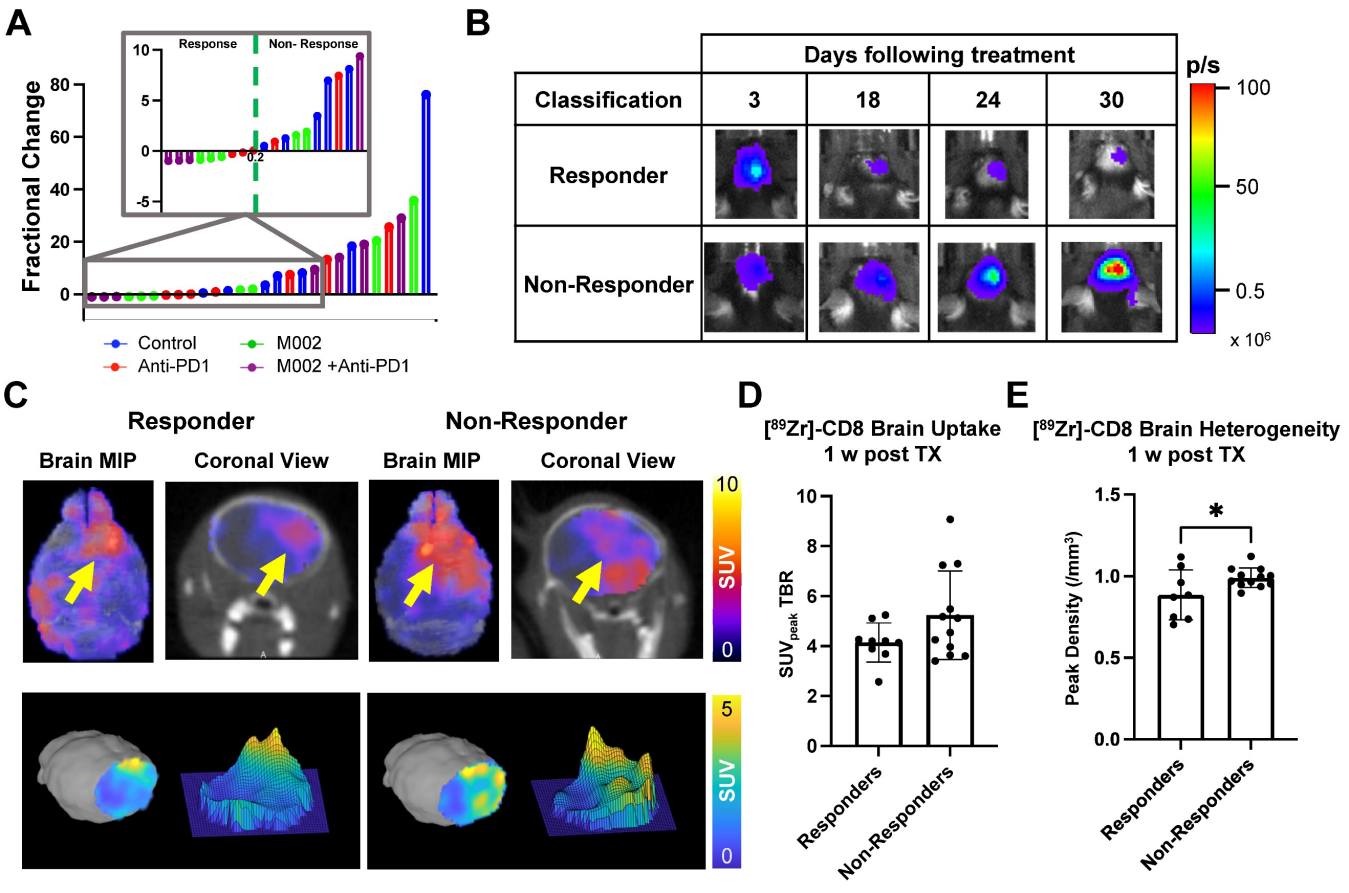
** [^89^Zr]-CD8 ImmunoPET imaging revealed differences in CD8 infiltration between responders and nonresponders one week following immunotherapy. (A)** Plot of endpoint fractional change of all treatment groups, response classification threshold was set as a 20% increase (> 0.2 fractional change) in tumor burden for non-responders. Of interest, under this classification, all responders belonged to an immunotherapy treated cohort (N = 3/7 for anti-PD1, M002 and combination). **(B)** Representative BLI scans of responders and non-responders following treatment administration. **(C)** Representative ImmunoPET/CT brain MIP, coronal cross-section (top row) and single brain slice signal distribution (bottom row) of a responder and a non-responder, yellow arrow highlights the signal accumulation at the tumor region.** (D)** Quantification of SUV_peak_ TBR (left) and heterogeneity (right), as indicated by regional peaks density, showed slight differences in CD8^+^ cell localization (p > 0.05) and decreased heterogeneity (p < 0.05) in immunotherapy responders relative to non-responders.

Bioluminescence imaging (BLI) was performed on a three-day basis following treatment administration to monitor changes in tumor viability, see Figure 3B for representative scans. Evaluation of viable tumor presence through BLI did not show any statistically significant changes across treatment groups at the initial [^89^Zr]-CD8 ImmunoPET imaging timepoint (D30) or one week following immunotherapy (p > 0.05), see Figures 3C. Response classification through BLI, defined as less than 20% increase in tumor burden over the course of evaluation, revealed an absence of responders in the control group (N = 0/7), while immunotherapy treated mice had a 42.9% response rate in all groups (N = 3/7 for anti-PD1, M002 and combination), as shown in Figure 4A.

Representative BLI and [^89^Zr]-CD8 PET/CT scans for responsive and non-responsive mice can be seen on Figure 4B-C. Within those classified as responders, a higher overall decrease in tumor burden was seen in M002 single agent (73.3 ± 15.0%, p < 0.01) and combination (94.3 ± 4.7%, p < 0.001) when compared to single agent anti-PD1 (12.6 ± 17.6%), see Figure S2D. Under this classification, a significant difference in tumor burden was seen between responders and non-responders two weeks following initial treatment (p < 0.05), which was preserved for the remaining BLI timepoints, see Figure S2A-B.

### Early [^89^Zr]-CD8 ImmunoPET imaging showed differences in CD8^+^ cell infiltration related to immunotherapy response

Based on response thresholding from long-term BLI changes, CD8^+^ cell infiltration in the brain was evaluated prior to changes in tumor burden via [^89^Zr]-CD8 ImmunoPET imaging. A trend towards increases in SUV_peak_ TBR, which highlights hotspots of CD8^+^ cells within the tumor region, was seen in nonresponders (5.23 ± 1.77) when compared to responders (4.15 ± 0.78) but did not reach significance (p > 0.05), Figure 4D. In addition, evaluation for the heterogeneity of CD8 signal distribution as measured by number of regional peaks over tumor region, see Figure S3, showed significantly decreased heterogeneity in immunotherapy responders (0.88 ± 0.15, p < 0.05) when compared to immunotherapy non-responders (0.99 ± 0.06), see Figure 4E and Table S2.

### Histological analysis showed correlation with [^89^Zr]-CD8 ImmunoPET, autoradiography, and bioluminescence imaging

**Figure 5:**
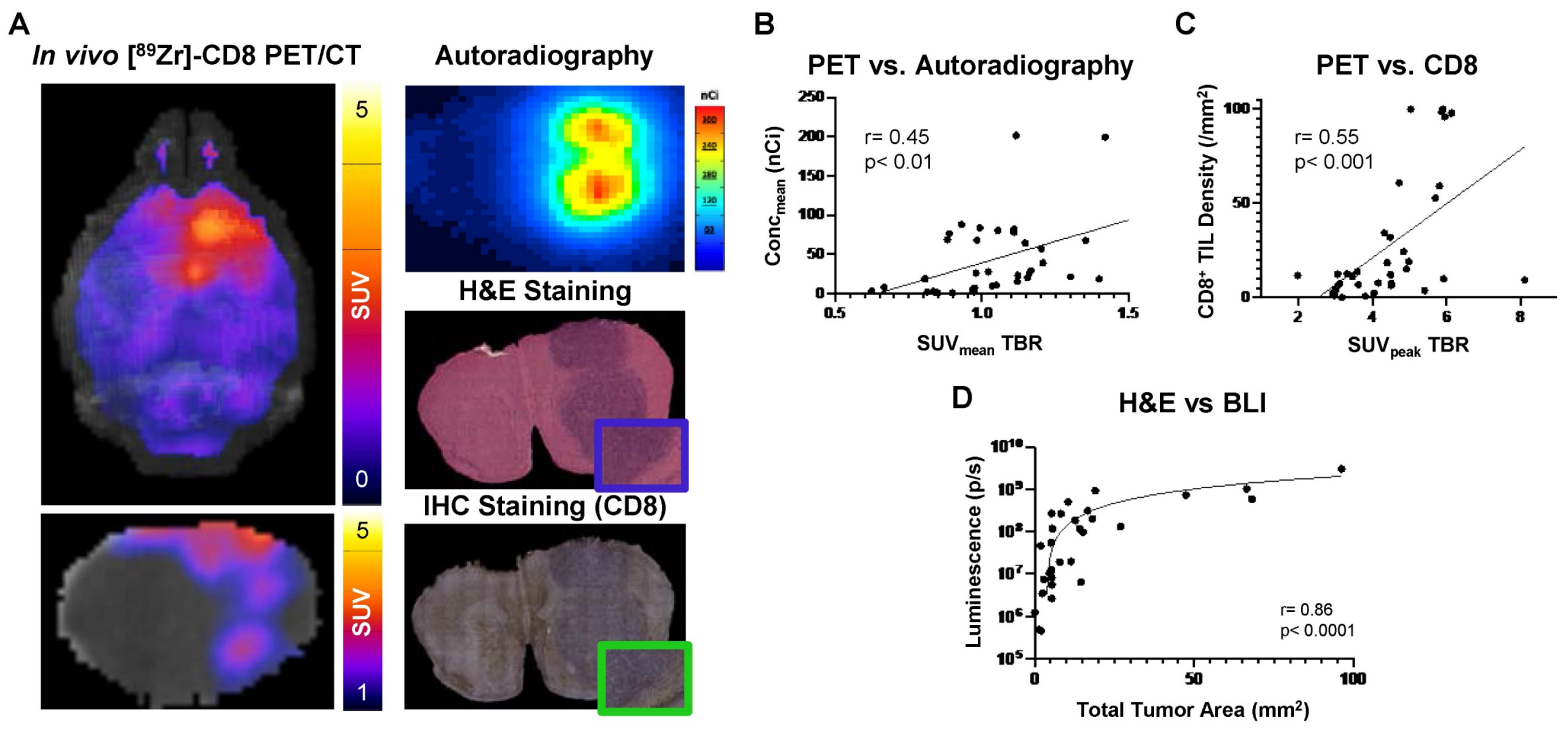
** Autoradiography and histological analysis showed correlations with [^89^Zr]-CD8 minibody ImmunoPET and BLI. (A)** Representative images for in vivo [^89^Zr]-CD8 ImmunoPET scan (left), autoradiography (top right) and H&E (mid right) and IHC for CD8 (bottom right) stained brain sections**. (B)** Linear regression of ImmunoPET SUV_mean_ TBR with recorded average brain radiotracer concentration (Conc_mean_) in autoradiography showed a positive correlation between these factors (r = 0.45, p < 0.01). **(C)** ImmunoPET SUV_peak_ TBR showed a positive correlation with IHC CD8^+^ tumor infiltrating lymphocyte (TIL) density (r = 0.55, p < 0.001). **(D)** Total H&E tumor area was strongly correlated with BLI signal intensity (r = 0.86, p < 0.0001).

Biological validation of [^89^Zr]-CD8 minibody ImmunoPET, autoradiography, and BLI was performed through histology for H&E and IHC staining for CD8. As shown on Figure 5A, tracer accumulation seen on both PET and autoradiography was primarily localized to the tumor region, as confirmed by H&E staining. Further, a positive correlation was seen between autoradiography brain concentration and* in vivo* [^89^Zr]-CD8 minibody ImmunoPET SUV_mean_ quantification (r = 0.45, p < 0.01), see Figure 5B. In addition, ImmunoPET metrics were positively correlated to intratumoral CD8^+^ cell infiltration (r = 0.55, p < 0.001), as shown by Figure 5C. Finally, BLI signal was confirmed to be indicative of tumor burden as shown by the strong positive correlation with total tumor area quantified from H&E staining (r = 0.86, p < 0.0001), see Figure 5D.

## Discussion

Immune-targeted PET imaging offers valuable information for the monitoring of infiltration and spatial distribution of immune cell populations in the glioblastoma tumor microenvironment during combination immunotherapy. In this study, we non-invasively evaluated early differences in CD8^+^ cell infiltration and their longitudinal effects associated with combination therapy oHSV and anti-PD1 via [^89^Zr]-CD8 ImmunoPET imaging in an orthotopic murine glioblastoma model. Our data demonstrated that combination M002 and anti-PD1 results in an increased infiltration of CD8^+^ cells over control mice in the tumor region, which is consistent with previously reported findings on combination oHSV and ICI immune promotion in preclinical GBM 14. These findings were further shown to be representative of CD8^+^ cellular localization into the GBM tumor microenvironment via *ex vivo* imaging, autoradiography, and histological analysis. Longitudinally, we found that a more homogeneous distribution of CD8^+^ cells in the brain tumor region one week following immunotherapy was associated with positive response to treatment. In the literature, past studies have highlighted the role of intratumoral effector T cells and their association to positive immunotherapy response in GBM 23, 27, while no assessment of immunological spatial heterogeneity has been previously reported. For the first time, our results provide information on GBM spatial CD8^+^ cell localization following immunotherapy and suggest methodologies for the evaluation of response using tumor signal heterogeneity.

As an immunotherapeutic approach for GBM, IL-12 expressing oHSVs have been shown to stimulate intratumoral infiltration of immune cells leading to tumor regression in murine preclinical models 9, 14, 35-37. These positive effects have been attributed to the multifaceted anti-tumoral properties of IL-12, including T_h_1 maturation of CD8^+^ T cells and induction of anti-angiogenic mechanisms 9, 38, in combination with adaptive immune cell recruitment and cancer cell lysis induced by oHSV therapy 8, 39. Our examination through *in vivo* [^89^Zr]-CD8 minibody ImmunoPET imaging did not show a significant change in intratumoral CD8^+^ cell infiltration, based on SUV_peak_ TBR, following single agent M002 oHSV one-week post-therapy, which differs from previously published literature on CD8-targeted cys-diabody PET imaging of M002 virotherapy in preclinical GBM 31. Further examination of *ex vivo* ImmunoPET imaging revealed a marked separation of responses in CD8^+^ cell recruitment in the M002 treated mice, which could be attributed to the differences in heterogeneity, immunosuppression, and vascularity of the GSC005 cell line, as well as its relative resistance to oHSV infection 4, 40. These tumor microenvironment changes would be further accentuated by the increased allotted time for tumor growth and development, totaling three weeks against conventional one week post implantation for treatment administration 14. These differences could lead to similar CD8^+^ cell levels to that of controls, previously reported in a study evaluating single agent IL-12 expressing oHSV in preclinical GBM 14. In contrast, combination of M002 and anti-PD1 immunotherapies resulted in a significant increase in CD8^+^ localization to the tumor microenvironment in both *in vivo* and *ex vivo* imaging, providing supportive evidence on the immunological benefit of combination oHSV and ICI previously reported in literature 14. Furthermore, as a single agent approach, anti-PD1 immunotherapy showed a limited increase in CD8^+^ cell immune promotion, which could be attributed to lack of CD8^+^ T cell presence in the tumor microenvironment 26, 41 and potential inability of the antibody to cross the blood brain barrier (BBB). The reported highly immunosuppressive nature of GBM and evidence of PD1 penetration in preclinical brain tumors provide supportive evidence for a more tumor microenvironmental influence 14, 42, 43. This limited immunological benefit of single agent anti-PD1 resembles clinical outcomes 3 and previously reported preclinical studies 14. Overall, our [^89^Zr]-CD8 minibody ImmunoPET imaging approach revealed differences in promotion of CD8^+^ cell infiltration to the GBM tumor microenvironment following M002, anti-PD1 and combination immunotherapy.

To further explore the effects of differential CD8^+^ immune cell infiltration in the GBM tumor microenvironment, we performed a longitudinal evaluation of immunotherapy response while monitoring via [^89^Zr]-CD8 ImmunoPET imaging one- and three-weeks post treatment. Interestingly, assessment of changes in CD8^+^ cell presence in the overall brain, as quantified by brain ImmunoPET SUV_mean_, revealed an increased localization of cells in all controls, while immunotherapy treated mice exhibited variability in between imaging timepoints. While literature related to associations between long-term immune recruitment and localization in the untreated preclinical GBM tumor microenvironment is limited, there have been studies suggesting a retained expression of pro-inflammatory cytokines, such as IFN-γ, in CD4^+^ cells promoting T cell migration into the CNS in GBM 44, 45. These infiltrating CD8^+^ lymphocytes have been primarily associated with inactivity and exhaustion given their constant exposure to tumor antigens and their high expression of inhibitory receptors such as PD-1, LAG-3, TIGIT, and CD39 46-48. In addition, natural increases in tumor volume within this timeframe could allow for nonspecific tracer accumulation under enhanced permeability and retention (EPR) effects 49. On the other hand, the variable changes seen in immunotherapy treated groups could be attributed to a similar effect coupled to immunological response or counteracted by a decreased tumor burden between timepoints, as seen on CD8-targeted PET imaging of responding tumors undergoing immunotherapy 50. Overall, these findings provide evidence on the changing immune GBM landscape in response to immunotherapy, as well as in the absence of treatment, that can be further explored for improvements in dose management and timing strategies for GBM.

Correlation of tumor size and BLI signal was confirmed in our histological analysis and in published literature 51, 52. Early in the course of therapy, we found that non-responders were associated with a more heterogeneous presence of CD8^+^ cells in the GBM tumor microenvironment. The trending increases in CD8^+^ cell presence in non-responder mice were unexpected given the cells' cytotoxic role in GBM. Evidence in the literature points to multiple factors influencing proper cytotoxic CD8^+^ T cell function including the recruitment of effector CD8^+^ T cell subpopulations (tumor antigen specific, granzyme B^+^, IFNγ^+^), reinvigoration of exhausted and inactivated CD8^+^ T cells (PD1^+^, LAG3^+^, TIM-3^+^, CD25^-^), and optimal immune microenvironment (increased CD8^+^ T cell / CD4^+^FoxP3^+^ T cell ratio, T_h_1 CD4^+^ T helper immune response, M1 macrophage phenotype) in GBM immunotherapy 23, 44, 53-57. These populational and microenvironmental influences needed for proper immunotherapy response could limit the ability of CD8 as a single biomarker for GBM imaging to properly elucidate on downstream response based solely on imaging.

Changes in CD8^+^ T cell distribution within the tumor microenvironment have been identified as a factor for proper immunotherapy response in multiple cancer models 58-61 with non-responsive lesions exhibiting a more peripheral and dispersed localization of this immune population. Given the heterogeneous, immunosuppressive nature of GBM and its documented influence on immunotherapy response 23, 56, 61, we hypothesized that heterogeneity in distribution of the CD8^+^ immune population in the brain would be associated with downstream therapeutic benefit. Therefore, based on methodology proposed by Rashidian et al. with a similar ImmunoPET imaging approach 24, 62, we evaluated the regional heterogeneity of CD8 expression in the brain. Through this approach, we found that responders were associated with a more homogeneous distribution relative to non-responders one-week following immunotherapy. While our evaluation was focused on whole brain distribution, our findings provide supportive evidence on the impact of the spatial distribution of CD8^+^ immune cell infiltration in positive GBM immunotherapy response. Evaluation of immune cell distribution and localization in the tumor microenvironment through these advanced PET approaches, has the potential to elucidate on early immunotherapy response and allow for early patient stratification for additional oHSV therapeutic doses or alternative adjuvant therapies.

While our findings on the impact of heterogeneity of CD8^+^ cell distribution in the tumor microenvironment can provide meaningful information on immune responses in GBM following immunotherapy, our study has various limitations. First, as optimal administration of oHSV requires intracranial injections, there is an expected immune response at the injury site from the orthotopic administration as reported in previous studies 31. To reduce the signal from the healing response from the invasive procedure and primarily focus on regions within the tumor implantation site, automated methods were used to remove the olfactory bulb and brain cortex regions. However, this approach could create a small potential for missing relevant tumor regions during analysis. Second, ImmunoPET CD8-targeting minibody is not solely specific to cytotoxic T cells, as other populations, such as dendritic cells, have been shown to express CD8 in mice 63. Future studies through flow cytometry could elucidate on the influence and presence of other CD8 expressing immune populations within the tumor microenvironment. However, this would require the use of invasive biopsies to properly associate this information with long-term response in preclinical models. Third, this imaging study was limited to a single syngeneic GBM cell line, which could limit the scope of the described approach. GSC005 was selected given its previously described variable response to single agent and combination oHSV and ICI 14, and a median survival that would allow for long-term imaging of both treated and untreated mice. These characteristics are challenging in other GBM models, such as 4C8, GL261 and CT2A being responsive to M002, responsive to ICI or having low median survival, respectively 14, 37, 64. Fourth, while BLI has been implemented for the evaluation of response in orthotopic preclinical brain tumor models 51, 52, 65, this response assessment approach would not be completely representative of the clinical standard of care for GBM, as iRANO uses anatomical MRI metrics for response assessment with potential for long-term follow-up imaging in case of early radiological progression 7. While assessment of pseudoprogression through anatomical MRI was beyond the scope of this study, future experiments could explore the combinatorial benefits of PET/MR in these preclinical models of immunotherapy response. Combination of these modalities would also allow the study of intratumoral and peripheral immune cell localization and distribution within the tumor microenvironment using similar immunotherapy-treated models. Further, CD8 ImmunoPET assessment of long-term immuno-surveillance in the context of recurrence, via GSC005 rechallenging, could further elucidate on positive immunological kinetics of immunotherapy response as past studies have shown strong immunological effects after rechallenging following virotherapy-induced tumor regression 14, 66. Finally, despite the conflicting evidence on the immune-promoting nature of luciferase expression on GBM cell lines 67, 68, we acknowledge the potential limitation of this model to not be fully representative of the highly immunosuppressive nature of GBM. Nevertheless, the absence of responders in the controls, and variable responses coupled with changes in immune cell dynamics in the immunotherapy groups provide evidence suggesting that the therapy-induced immunological changes resulted in downstream decreased tumor burden.

In conclusion, this study provided evidence on the importance of monitoring immune cell dynamics and distribution in the tumor microenvironment for the assessment of immunotherapy-induced effects in GBM. Immunotherapeutic response was primarily associated with a sustained homogeneous distribution of CD8^+^ immune cells recruited to the brain, while there was a dissociation between increased CD8^+^ localization and subsequent therapeutic benefit. The longitudinal implementation of this sensitive and specific ImmunoPET imaging method provides meaningful information on the CD8^+^ immune population which has the potential to inform on immunotherapeutic benefits and subsequent response.

## Materials and Methods

### CD8^+^ cell infiltration assessment

#### Tumor Development and Monitoring

GSC005-luc positive cells (5x10^5^), gifted by Dr. Inder Verma of the Salk Lake Institute, were implanted intracranially on the right hemisphere of 5-6-week-old female C57BL/6 mice (N = 40, Charles River Laboratories, Wilmington, MA) at day 0. Tumor growth was monitored through BLI with IVIS Lumina III (Perkin Elmer, Waltham, MA) on days 10, 17 and 24 post implantations. Mice received an intraperitoneal injection of D-luciferin (115144-35-9, GoldBio, Olivette, MO), which was allowed to circulate for a total of 10 minutes, followed by a 5-minute bioluminescence acquisition. Quantification of total flux (p/s) was performed in the cranial region of the mice, as well as an individual flanks for assessment of background signal, with a standardized sized circular region of interest (ROI) across all measurements using Living Image (Waltham, MA). Mice with a tenfold increase in quantified signal in comparison to background were categorized as tumor positive and enrolled into the study.

#### Bioluminescence Imaging Analysis and Response Assessment

BLI was performed, using the same approach previously described for tumor growth monitoring, every three days following treatment for the assessment of response. Fractional change was defined as the difference between the signal at a given timepoint and baseline (D25), divided by baseline. Responders were thresholded based on an increase of less than 20% or 0.2 fraction change from baseline, while non-responders encompassed those with a greater than 20% increase in tumor signal. Threshold was based on iRANO defined metrics 18, while assessment was performed on viable cell signal rather than MRI-defined dimensions, with responders encompassing stable, and partial and complete response.

#### Treatment Administration

Mice were randomly assorted, through a computational randomization algorithm 69 with reassortment being performed until statistical analysis corroborated an absence of significance differences in baseline BLI signal, see Figure S2C, into four cohorts based on treatment: saline (N = 9), single agent M002 oHSV (N = 11), single agent anti-PD1 (N = 6), and combination of M002 and anti-PD1 (N = 8). Intratumoral injection of M002 oHSV at a dose of 2x10^7^ plaque forming units (pfu)/ 2μl was administered for mice in the M002 and combination groups while the remaining cohorts received 2 μl of saline on day 25. M002 dosing was increased from published literature 31 as to allow for a more robust T-cell response. Anti-mouse PD1 (RMP1-14, Bio X Cell, Lebanon, NH) was administered via intraperitoneal injection at a dose of 10 mg/kg for the single agent anti-PD1 and combination groups on days 26, 28 and 30. Longitudinal response assessment was performed with similar treatment groups (N = 7 per group) with equivalent treatment dosing while timing for anti-PD1 administration was modified to day 26, 29 and 32.

#### [^89^Zr]-DFO-CD8 radiolabeling

Anti-mouse CD8 minibody (Df-IAB42, ImaginAb, Inglewood, CA) was conjugated with deferoxamine (DFO) chelator and radiolabeled with [^89^Zr]-oxalate, provided by UAB cyclotron, based on a previously defined protocol with slight modifications 31. In short, [^89^Zr]-oxalate was diluted with equal volume of 1M HEPES followed by 2M NaOH solution until a pH of 7.02 ± 0.2 was achieved. Conjugated minibody was then added at predetermined concentration of 10 µCi/µg, as obtained by a labeling efficiency study, see Figure S4A, and mixed at 500 RPM at 37 °C for one hour using a thermomixer. This was then purified using 7 K MWCO Zeba Spin Desalting Columns (Thermo Fisher, Waltham, MA) washed with phosphate buffered saline (PBS). The purity of the radiolabeled tracer was determined using instant thin layer chromatography (iTLC) with 50 mM diethylenetriamine pentaacetate (DTPA) as developing agent 70. Further, stability of radiolabeling was tested in mouse serum (N = 3) and PBS (N = 3) dilutions with purity checks at 0, 1, 4, 12, 24, 72 and 168 hours post labeling via iTLC corroborating radiolabel stability at the imaging time point, see Figure S4B.

#### PET/CT Imaging

[^89^Zr]-DFO-CD8 minibody was injected at a dose of 90 ± 10 μCi intravenously via tail vein on D30. PET (energy window 350-650 keV; 20 min static) and CT (voltage 80 kVp; current 150 μCi; 720 projections) were acquired in vivo 24 hours post injection using a GNEXT small animal PET/CT imaging system (Sofie Bioscience, Culver City, CA). Mice were then humanely euthanized, and brains were extracted for ex vivo PET (one hour static) and CT (80 kVp) acquisitions. Brains were then placed in formalin and fixed overnight for autoradiography and IHC analysis.

For analysis, PET and CT scans were registered, and a brain ROI was automatically generated using VivoQuant's (Invicro, Boston, MA) Brain Atlas Tool and Otsu's segmentation algorithm on CT for *in vivo* and *ex vivo* scans, respectively. Brain ROIs were redefined by removing the olfactory bulbs and cortex based on the Brain Atlas Tool provided regions, representative schematics can be seen on Figure S1A-C. Mean, standard deviation, peak and max values for standardized uptake value (SUV) were quantified based on the formula 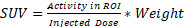. Additional evaluation of tumor to background ratios (TBR) was performed by quantifying SUV_mean_, SUV_max_ and SUV_peak_ on the right brain hemisphere and normalizing them to the contralateral brain side. Finally, heterogeneity analysis was performed by isolating the brain PET region based on the previously defined ROI using VivoQuant and performing an automated MATLAB R2022 (Mathworks, Natick, MA) algorithm to identify regions of peak concentration within the scan. This method consists of generating a mask of regional peaks on a slice-by-slice basis, using the imregionalmax function, and quantifying the peaks as volumetric regions, through the function regionprops3 with maximum connectivity. Peak density (/mm^3^) was defined as the total number of quantified peaks divided by total volume analyzed, acquired by multiplying the number of voxels by their real-world dimensions.

### Biological validation assays

#### Autoradiography

Following *ex vivo* imaging, brains were coronally sliced into approximately twelve 1mm sections using a rodent brain matrix (Braintree Scientific, Braintree, MA) and placed on an exposure cassette (GE Healthcare, Chicago, IL) along with 1 nCi, 2 nCi and 10 nCi standards. Film exposure was performed for a total of 3 hours and acquired using an Amersham Typhoon laser-scanner (Cytiva, Little Chalfont, UK). Autoradiography scans were then calibrated and quantified using Vivoquant's Autoradiography Calibration Tool. ROIs of the tumor sections were semi-automatically generated for each brain slice using global thresholding and regional smoothing. Brain slices were segmented into high signal, defined as tumor, and low signal, defined as non-tumor, using optimal thresholding. Percent tumor being was defined as the division of the number of pixels of high and low signal regions. In addition, mean, max and peak autoradiography concentrations (Conc) were calculated for the combination of individual brain sections.

#### Histology staining

Two central brain slices with the highest radiotracer concentration from autoradiography, as given by autoradiography Conc_mean_, per subject were selected for histological analysis. Paraffin embedding, slicing into 5 μm sections and staining for hematoxylin and eosin (H&E) procedures were performed by UAB histology core facility 71. Slices were dewaxed using EZ-DEWAX (HK584, BioGenex, Fremont, CA) twice for 5 minutes, followed by antigen retrieval with citrate buffer using an EZ-Retriever System (MW015-IR, BioGenex, Fremont, CA) and blocking with 5% BSA in TBST for 5 minutes at room temperature. CD8a monoclonal primary antibody (1:100, 4SM15, Invitrogen, Waltham, MA) was incubated overnight at 4 ºC. Anti-Rat-Ig HRP Detection kit (51-7605KC, BD Pharmingen, Franklin Lakes, NJ) was used for the secondary antibody (1:100). DAB substrate (SK-4100, Vector Laboratories, Newark, CA) was incubated for 7 minutes at room temperature for the development of the stain. Scanning of H&E and IHC stained slides was performed using an EVOS M7000 Imaging System (Thermo Fisher, Waltham, MA) at 20x magnification.

#### Histology quantification

Custom MATLAB code was developed for the analysis of H&E tumor area and positive CD8 staining. Automatic H&E classification of the region of high cellular density was performed using a four-cluster k-means clustering segmentation method, which generated a binary mask of the tumor. Summation of pixels in the tumor mask provided the total tumor area, measured in mm^2^ as provided by the scanner resolution, which was then divided by the total brain pixels for evaluation of tumor percentage. Total tumor area (mm^2^) was defined as the sum of the tumor areas in the four representative sections acquired for the longitudinal assessment of immunotherapy response. Positive CD8 cell quantification in the tumor region was performed using semiautomated MATLAB algorithms. Tumor region was manually delineated, based on the H&E defined region, and evaluated for presence of positively stained cells using double optimal thresholding on the color saturation metric. Number of positively stained cells was obtained by counting the number of objects with high color saturation. Tumor infiltrating lymphocyte (TIL) density was defined as the total number of CD8^+^ IHC cells divided by the total tumor area (/mm^2^).

#### Statistical analysis

Statistical evaluations were performed using Prism 9 (GraphPad Software, San Diego, CA). Comparisons to control groups were performed using ordinary one-way ANOVA with Dunnett's test for Figures 2B-D, 3E, and S2D. Further, comparisons between groups were performed using one-way ANOVA with Tukey's correction for multiple comparisons for supplemental Figure 2C. Unpaired two-tailed t tests were conducted for the analysis of Figures 4D-E. Pearson's correlation tests were conducted in Figures 2E, and 5B-D. Statistical outliers were identified and removed using Grubb's test for outliers with an alpha of 0.05 as follows: two for Figure 2B (N = 1 control, N = 1 anti-PD1), three for Figure 2D (N = 2 control, N = 1 combination), one for Figure 3E (N = 1 M002), and one for Figure 4E (N = 1 non-responders).

## Supplementary Material

Supplementary figures and tables.
