# Supplementary material for: [89Zr]-CD8 ImmunoPET imaging of glioblastoma multiforme response to combination oncolytic viral and checkpoint inhibitor immunotherapy reveals CD8 infiltration differential changes in preclinical models
Source: Theranostics. 2024 Jan 1;14(3):911–23. doi: 10.7150/thno.89206 (PMC10797292; doi:10.7150/thno.89206)
Supplement: Supplementary file 1 — Supplementary figures and tables. [file thnov14p0911s1.pdf]

SUPPLEMENTAL

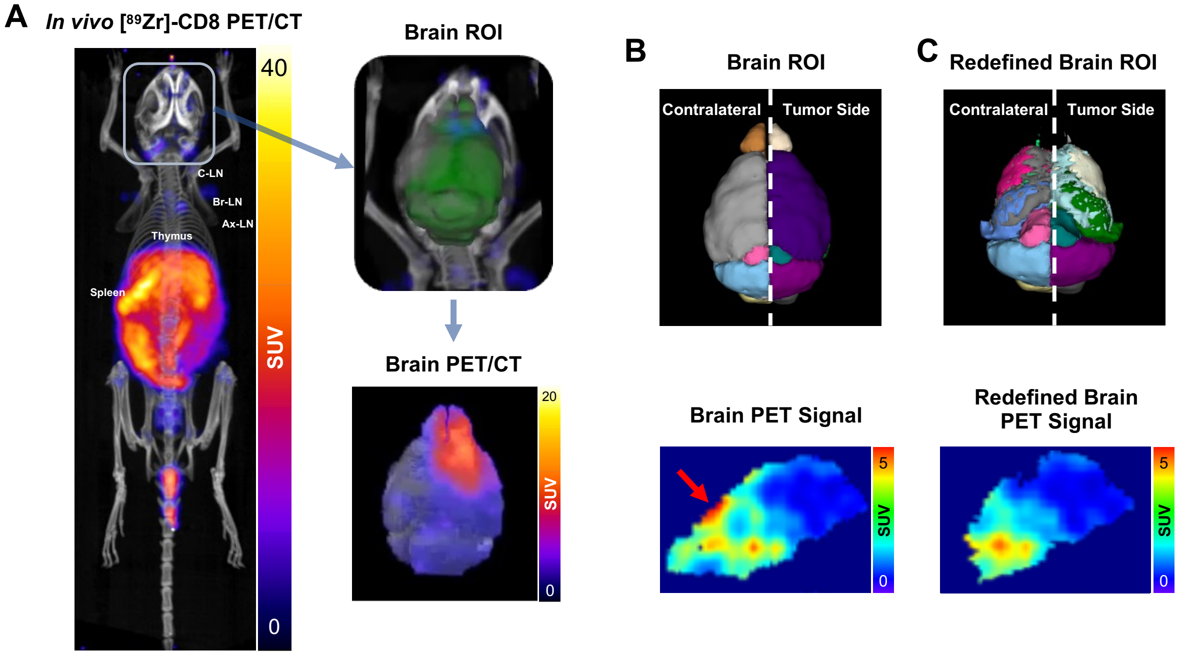

Figure S1. PET/CT Processing Workflow. (A) Representative full body in vivo [^{89}Zr]-CD8 minibody ImmunoPET/CT scan (left) showed accumulation in immune associated organs including spleen, thymus, and lymph nodes. 3D render of brain defined ROI (top right) was automatically generated for *in vivo* SUV uptake quantification and brain isolation for quantification (bottom right). **(B)** Representative 3D rendering of brain subregions, white line represents division of tumor and contralateral tumor side defined for quantification. Visualization of tracer uptake through a sagittal view (bottom) showed a region of tracer accumulation associated with inflammation from intracranial injury from treatment as highlighted by the red arrow. **(C)** Representative 3D rendering of redefined brain ROI with the removal of the olfactory bulb and cortex (top). Through this approach, influence of CD8^{+} cell localization associated from inflammation is removed from analysis as seen on the representative sagittal slice (bottom). C-LN: Cervical Lymph nodes, BR-LN: Branchial Lymph Nodes, Ax-LN: Axillary Lymph Nodes, ROI: Region of Interest, SUV: Standardized Uptake Value.

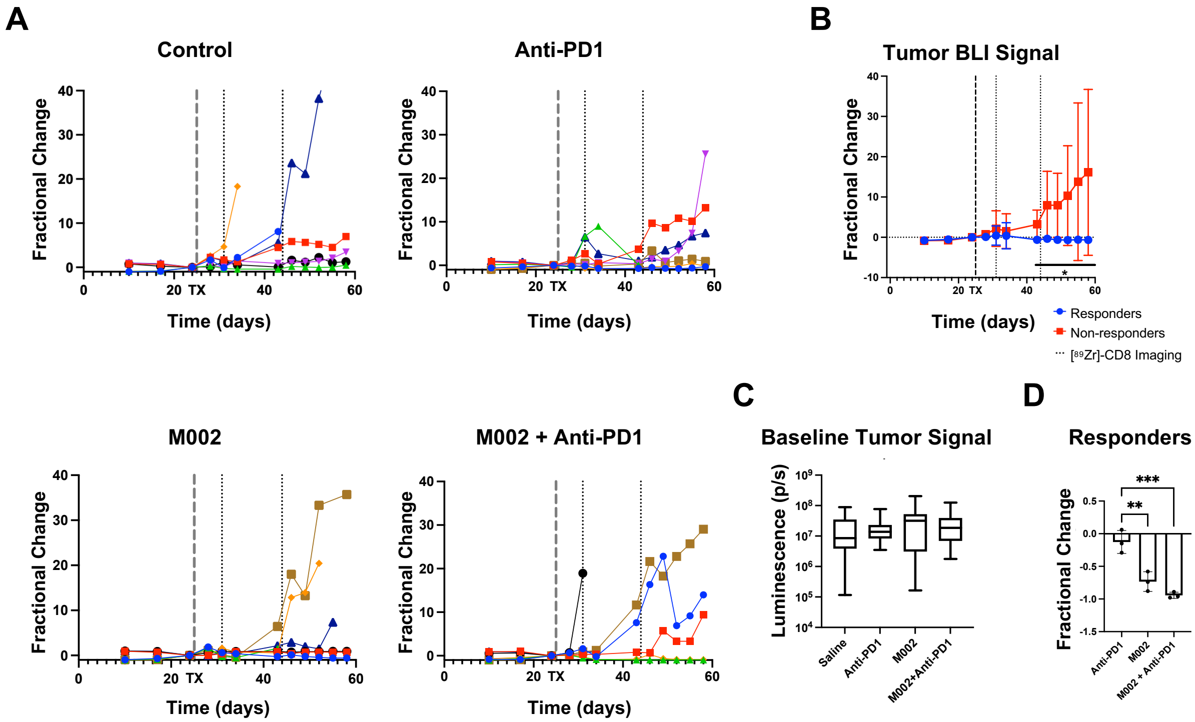

Figure S2. Bioluminescence imaging revealed longitudinal variability within treatment groups. A) Fractional change of mean tumor BLI signal for individual mice of each treatment group: control (top left), anti-PD1 (top right), M002 (bottom left) and combination immunotherapy (bottom right). **(B)** Fractional changes from baseline of mean tumor BLI signal for responders and non-responders revealed significant difference in tumor burden between responders and non-responders ($p < 0.05$). **(C)** Baseline tumor BLI signal showed no significant differences in tumor burden across treatment groups ($p < 0.05$) **(D)** Endpoint fractional change of mean BLI signal for immunotherapy responders showed a significant decrease in tumor burden in M002 ($p < 0.01$) and combination ($p < 0.001$) treated groups when compared to single agent anti-PD1.

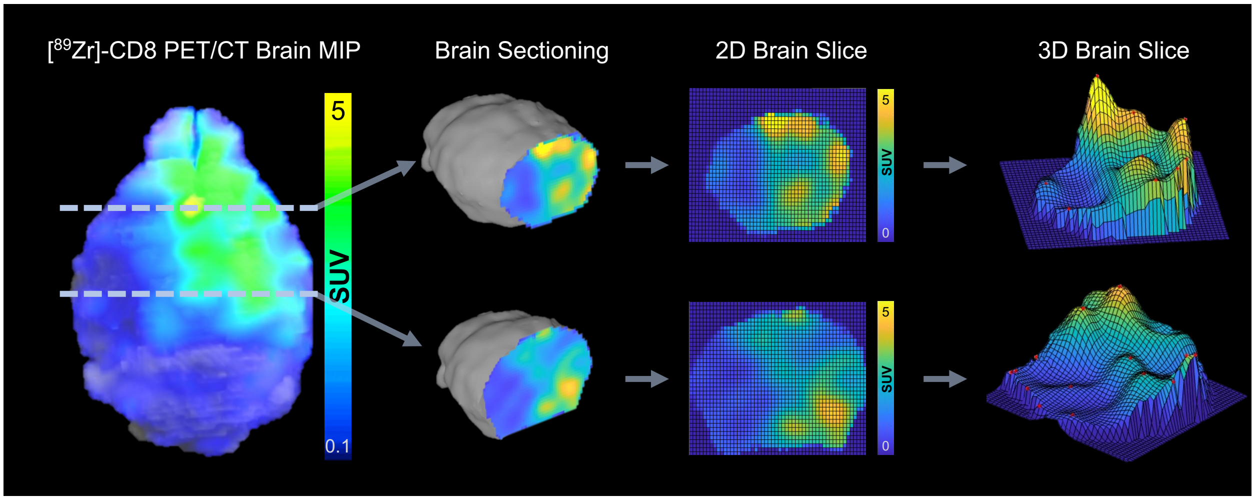

Figure S3. Heterogeneity Processing Workflow. Analysis of regional peaks was performed on a slice-by-slice basis across the brain defined region. Process involved isolating individual 2D PET signal slices (brain sectioning) and determining regional peaks through automated methods, as shown on the 2D and 3D representative brain slices.

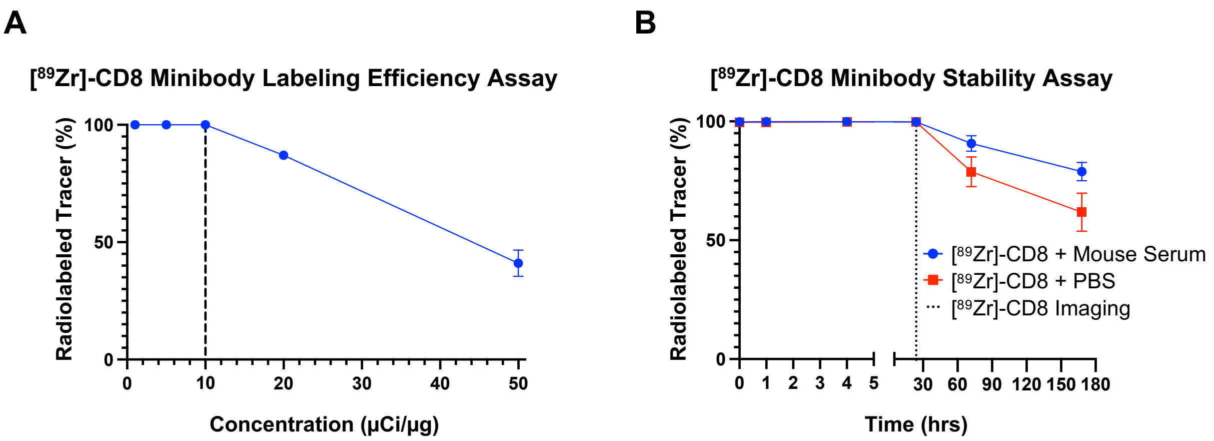

Figure S4. [^{89}Zr]-CD8 Minibody Radiolabeling Optimization and Validation. (A) Labeling efficiency assay of various radioisotope and minibody concentrations used for the selection of optimal labeling concentration of 10 μCi/ μg (dotted line). **(B)** Longitudinal stability assay corroborated radiolabeling stability of [^{89}Zr]-CD8 minibody tracer in mouse serum and PBS at imaging timepoint (24 hours) and up to seven days following initial radiolabeling.

Table S1. Summarized numerical data (mean ± standard deviation) associated with imaging quantification of [^{89}Zr]-CD8 PET for treatment cohorts.

	Treatment Cohort			
**Metric**	**Saline**	**Anti-PD1**	**M002**	**M002 + Anti-PD1**
In vivo Brain SUV_{peak} TBR	3.46 ± 0.77	4.41 ± 0.70	4.10 ± 1.07	5.29 ± 1.66
In vivo Cervical LN SUV_{peakr}	5.26 ± 1.27	6.01 ± 1.20	6.93 ± 1.34	6.51 ± 1.42
Ex vivo Brain SUV_{peak}	0.75 ± 0.30	1.19 ± 0.47	1.28 ± 0.63	1.60 ± 0.22
In vivo Brain SUV_{mean} Change	0.58 ± 0.39	0.07 ± 0.44	-0.16 ± 0.21	0.22 ± 0.68

Table S2. Summarized numerical data (mean ± standard deviation) associated with imaging quantification of *in vivo* brain [^{89}Zr]-CD8 PET based on response classification.

	Response Cohort	
**Metric**	**Responders**	**Non-responders**
SUV_{peak} TBR	4.15 ± 0.78	5.23 ± 1.77
Peak Density	0.89 ± 0.15	0.99 ± 0.06
